# Can Implementation of Genetics and Pharmacogenomics Improve Treatment of Chronic Low Back Pain?

**DOI:** 10.3390/pharmaceutics12090894

**Published:** 2020-09-21

**Authors:** Vladislav Suntsov, Filip Jovanovic, Emilija Knezevic, Kenneth D. Candido, Nebojsa Nick Knezevic

**Affiliations:** 1 Department of Anesthesiology, Advocate Illinois Masonic Medical Center, 836 W. Wellington Ave. Suite 4815, Chicago, IL 60657, USA; vladislav.suntsov@aah.org (V.S.); drfilipjovanovic91@gmail.com (F.J.); ekneze2@illinois.edu (E.K.); kenneth.candido@aah.org (K.D.C.); 2 Department of Anesthesiology, University of Illinois, Chicago, IL 60612, USA; 3 Department of Surgery, University of Illinois, Chicago, IL 60612, USA

**Keywords:** chronic low back pain (cLBP), genetics, pharmacogenomics, personalized treatment, polymorphism, CYP450

## Abstract

Etiology of back pain is multifactorial and not completely understood, and for the majority of people who suffer from chronic low back pain (cLBP), the precise cause cannot be determined. We know that back pain is somewhat heritable, chronic pain more so than acute. The aim of this review is to compile the genes identified by numerous genetic association studies of chronic pain conditions, focusing on cLBP specifically. Higher-order neurologic processes involved in pain maintenance and generation may explain genetic contributions and functional predisposition to formation of cLBP that does not involve spine pathology. Several genes have been identified in genetic association studies of cLBP and roughly, these genes could be grouped into several categories, coding for: receptors, enzymes, cytokines and related molecules, and transcription factors. Treatment of cLBP should be multimodal. In this review, we discuss how an individual’s genotype could affect their response to therapy, as well as how genetic polymorphisms in CYP450 and other enzymes are crucial for affecting the metabolic profile of drugs used for the treatment of cLBP. Implementation of gene-focused pharmacotherapy has the potential to deliver select, more efficacious drugs and avoid unnecessary, polypharmacy-related adverse events in many painful conditions, including cLBP.

## 1. Introduction

Low back pain (LBP) is an extremely common problem affecting 80% of individuals at some point in their lifetime. It is the fifth most common motive for all physician visits. A lifetime prevalence of LBP was found to be about 40% worldwide [1]. In the United States (US), LBP and related costs are escalating [1], along with many modalities and their application in managing this problem. Five to ten percent of patients will develop constant back pain. Chronic low back pain (cLBP) has a strong impact on society. The US Burden of Disease Collaborators have shown that in 1990 and 2010, LBP was a disability that persistently affected people for the longest amount of time.

From the 1990s to 2000s, healthcare costs for adults with spinal problems continuously increased, with a rough estimate of 6000 USD per person with cLBP in 2005, totaling 102 billion USD [2]. In the US, adults suffering from cLBP were found to make more frequent healthcare visits usually covered by government-sponsored health insurance plans and to be more socioeconomically disadvantaged [2].

The etiology of back pain is multifaceted and not completely understood. We know that back pain is somewhat heritable, chronic pain more so than acute. In as many as 80% of people suffering from cLBP, the precise cause cannot be determined. Despite cLBP often being connected to anatomic perturbations such as herniation or degeneration of the intervertebral disc (IVD), these physical findings have a weak association with cLBP [3,4] and account for only a fraction (7–23%) of the genetic influence on back pain [5]. Conversely, objective findings such as degenerative findings on imaging often do not translate into chronicity of LBP. Higher-order neurologic processes involved in pain maintenance and generation may explain genetic contributions and functional predisposition to the development of cLBP that does not involve spine pathology [6,7,8].

Several genes have been identified in genetic association studies of chronic pain conditions. The results of the mentioned studies suggest a pathophysiology based on disruption of tissue remodeling, with abundant pro-inflammatory signaling leading to pain [9]. In the following review, we compiled the genes identified by numerous genetic association studies with chronic pain conditions, focusing on LBP specifically. Roughly, these genes group into several categories, coding for: receptors, enzymes, cytokines and related molecules, opioid receptor ligands, and transcription factors.

## 2. Materials and Methods

We reviewed genetic association studies by conducting a keyword search on the PubMed database. The search keywords included: “chronic back pain”, “low back pain” combined with “genetics”, “genetic association”, “variant”, or “polymorphism”. Publications were screened by title and abstract. If the screening presented incomplete information, the text and tables/figures of the relevant publication were read and examined. We excluded reviews and publications that reported equivalent results from the same cohort. Barring several large populations studies, the bulk of the studies conducted presently have been done on modest population samples containing fewer than 1000 individuals.

## 3. Genes of Interest

### 3.1. Receptors

#### 3.1.1. OPRM1 (Opioid Receptor Mu 1)

OPRM1 is a gene coding for the mu (μ) opioid receptor, which is the primary target of opioid analgesics as well as endogenous opioid peptides (e.g., beta-endorphin and enkephalins). The mu-opioid receptor also has an important role in modulation of the dopamine system and subsequently, dependence on drugs of abuse, e.g., as nicotine, cocaine, and alcohol. Hasvik et al. [10] explored the relationship between the OPRM1 genotype and subjective health complaints (SHC) in patients with disc herniation and radicular pain. The Subjective Health Complaints Inventory was used as the primary outcome. The inventory includes 27 prevalent complaints experienced in the month prior and rated on a scale from ‘not at all’ (0) to ‘severe’ (3) [10]. Twenty-three out of 118 patients carried the OPRM1 G-allele. Single nucleotide polymorphism (SNP) genotyping was performed on the OPRM1 A118G. Females that carried the G-allele reported a decrease in pain at the one-year follow-up. When asked for pain scores and pain duration, female carriers had consistently more health complaints than male carriers throughout the study. Thus, the study surmised that in patients with radicular pain, SHCs are associated with sex, as seen through OPRM1 A118G polymorphism interaction [10]. Although it was formerly thought that the increased SHC was secondary to pain, these results suggested it might be more significant [11]. The interaction between sex and the OPRM1 polymorphism observed in this study confirms earlier findings. Reports state that μ-opioid receptor binding potential could be greater in women and with increasing age [12]. One study demonstrated region-specific divergence in levels of OPRM1 between individuals with AA and G alleles [13]. The *OPRM1* genotype may impart sensitivity to pro-inflammatory, immune, and stress responses [14], and sensitivity to social rejection [15]. Acute and chronic stress affects μ-opioid receptors in GABAergic neurons differently in male and female rats [16]. This occurs by a mechanism that is not understood.

#### 3.1.2. HTR2A (5-Hydroxytryptamine Receptor 2A)

Research has shown the associations between being susceptible to chronic pain conditions (e.g., chronic widespread pain and fibromyalgia) and serotonin receptor 2A (HTR2A) gene polymorphisms [17]. HTR2A gene polymorphisms rs6311 and rs6313 were found to be associated with higher disability, as measured by ODI (Oswestry Disability index) [18]. Polymorphism rs6311 (1438 A/G) was associated with chronic LBP, but patients with genotypes AA and AG had greater ODI scores [18]. Likewise, patients with TT or TC genotypes in rs6313 (102 T/C) polymorphism had higher ODI scores, but these genotypes were not associated with cLBP [18]. In an animal model, injection of exogenous 5-HT to the nerve root caused pain-associated findings, thus illustrating the role 5-HT plays in the initial biochemical pathogenesis of sciatic pain [19]. Moreover, selective serotonin reuptake inhibitors (SSRIs) have seen successful use in the treatment of cLBP. In a 2003 study by Kanayama et al., 300 mg of sarpogrelate hydrochloride, which is a selective 5-HT(2A) receptor blocker, was given orally for two weeks to 44 patients with symptomatic lumbar disc herniation. Visual analog scale (VAS) of LBP, numbness, and sciatic pain significantly improved post treatment with the serotonin receptor blocker, with >50% pain relief in 23 patients, 25–50% relief in five patients, and <25% relief in 16 patients. The effects of the 5HT2A receptor blocker saw more favorable response in patients with uncontained disc herniation than in patients with contained disc herniation [20].

#### 3.1.3. DCC (Deleted in Colorectal Carcinoma)

Another significant CBP-associated gene variant is the lead SNP rs4384683, an intronic variant in the gene DCC (deleted in colorectal carcinoma) [21]. Netrin-1 is an axonal guidance molecule, and as such, participates in the development of cortical and spinal commissural neurons. DCC encodes the protein that serves as a receptor for Netrin-1 [22]. DCC–Netrin-1 interactions are a well-studied axonal guidance mechanism that affects angiogenesis and are vital during development and adulthood [23,24]. Compared to healthy human IVDs, expression of both these genes is greater in degraded discs. They are also found less frequently in the annulus fibrosus than in the nucleus pulposus [25]. Neurovascular ingrowth into the IVD may be mediated by netrin-1 and DCC, which is a mechanism that has long been implicated in chronic discogenic back pain [25,26]. Given the phenotypic correlation between CBP and depression [27], the correlation between CBP and DCC (depressive symptoms associated with cross phenotype of rs4384683) could also be explained by pleiotropy [21]. In animal models of mechanical allodynia, interactions of Netrin1/DCC have been found to impact pain processing in the spinal cord [23]. In accord, these data suggest numerous possible causes for the relationship between CBP and DCC, including the involvement of mood and/or nociceptive pathways [21]. rs4384683 in the DCC gene was also associated with depressive symptoms [28] with the same trend i.e., the A allele was associated with lower risk of CBP.

#### 3.1.4. ESR (Estrogen Receptor 1)

Roh et al. [29] examined the relationship between estrogen receptor (ER) alpha (α) (ERα) polymorphisms and degenerative spondylolisthesis (DS) patients. A strong association was found between Xbal polymorphism and the VAS score of back pain. Subjects with AG and AA genotypes had significantly lower back pain (*p* < 0.05) VAS scores than did patients with a GG genotype. Identification of the CG haplotype with Pvull and Xbal polymorphism analysis in patients with back pain showed increased pain intensity on the VAS scale. ERα, a steroid hormone nuclear receptor, transactivates estrogen-responsive elements. Estrogen receptors are classified as ERα or ER beta (β), based on the mode of alternate gene splicing. The two receptors are significant regulators of skeletal maturation and growth [30,31]. The relationship between ERα and osteoarthritis has been recognized in a number of studies [32]. Thus, a gene in any part of the estrogen endocrine pathway is of interest to research in the pathogenesis of degenerative spondylolisthesis and broader implications for LBP.

#### 3.1.5. CNR2 (Cannabinoid Receptor 2)

The CNR2 receptor system is dynamically involved in pain processing. The current hypothesis is that following pain induction, the functional upregulation of spinal CNR2 protein and mRNA seems to contribute an important countermeasure to the formation of central sensitization. This is corroborated by the exacerbation of allodynia at the painful site, and the novel manifestation of allodynia in the control site in mice with genetically deleted *Cnr2* (*Cnr2^−/−^*) [33]. In a study by Ramesh et al., CNR2 mRNA expression was increased among patients with both acute and chronic LBP at baseline compared to healthy controls [34].

#### 3.1.6. ADRB2 (Adrenoceptor Beta 2)

Correlation was shown between SNP rs2053044 (ADRB2, recessive model) and CDCP (chronic disabling comorbid neck and low back pain). The study strongly suggests that genetic variants in the ADRB2 gene coding for the beta-2-adrenergic receptor makes individuals predisposed to chronic musculoskeletal complaints [35].

Some relevant receptor-related gene studies that are not referenced in the text are listed in Table 1.

### 3.2. Enzymes

#### 3.2.1. COMT (Catechol-O-Methyltransferase)

Catechol-O-Methyltransferase (COMT) is an enzyme that helps regulate adrenergic, nonadrenergic, and dopaminergic signaling through metabolizing catecholamines. Research has been done on a number of human and animal pain models to investigate the effects of decreased COMT enzyme activity on nociception. Peripheral pain sensitivity was found to be increased by low COMT activity in animal model data [41]. Low COMT activity in humans, however, attenuated spinal nociceptive activity and central sensitization [42]. Thus, it can be concluded that low COMT activity has a complex effect. A correlation between pain hypersensitivity and Met alleles producing low enzyme activity was often found in human pain models [43]. Pain sensitivity has been associated with a functional polymorphism reducing the enzyme activity in the gene encoding COMT, the COMT Val158Met SNP. Jacobsen et al. [44] examined COMT Val158Met SNP contribution to sciatica and discogenic subacute LBP. Degenerative disc disease (DDD) subjects’ appearance of the Val158Met genotypes was measured against healthy controls. It was hoped that this SNP may help in predicting the advancement of pain and disability. There were no differences in the frequency of the COMT genotype between controls and newly diagnosed subjects. When patients’ pain and disability were examined over time, a borderline significant rise in functionality measured with the ODI score and the McGill sensory score was found for patients who had a COMT Met/Met genotype. Furthermore, six months after inclusion, a significant relationship was observed between patients’ COMT Met-allele, pain (VAS score), McGill sensory, and ODI scores. It was also found that Val158Met SNP may contribute to disc herniation symptoms; patients with Met/Met had the slowest recovery and most pain, followed by those with Val/Met, followed by those with Val/Val [44]. Baseline disability was found to be significantly related to two haplotypes (*p* < 0.002), age, sex, and smoking (*p* ≤ 0.002), COMT SNPs rs6269 (*p* = 0.007), rs2075507 (*p* = 0.009), rs4818 in European adults (*p* = 0.02), and rs4633 (*p* = 0.04). There were no meaningful associations observed with clinical variables during the long-term follow up. Although this suggests that genetics plays a role in disability level in chronic LBP patients being considered for surgery, it was concluded that genetics does not affect the outcome of treatment in the long term [40]. A relationship between pain perception after lumbar discectomy and genetic polymorphism of the COMT enzyme was found by Rut et al. All of the subjects had a one-level symptomatic disc herniation from L3 to S1. The study tracked ODI to assess pain intensity and the patients’ quality of life, as well as VAS to assess back and leg pain. At the one-year follow-up, patients with the rs4680 GG genotype and COMT rs4633 CC demonstrated significant improvement in LBP. Better clinical outcome was shown in ODI scores and VAS for patients with COMT haplotype related to low metabolic activity of the enzyme (A_C_C_G) after surgery. It is noted that the study was too small to draw conclusions about the relationship between genetic diversity in COMT and clinical outcome after lumbar discectomy. It is suggested by the authors that the COMT genotype could serve a purpose in determining which patients would benefit more from surgery e.g., selection of subjects for earlier surgery [45].

#### 3.2.2. CASP9 (Caspase-9)

Caspase-9 (CASP-9) initiates apoptosis through signaling with the initiator caspase. CASP-9 influences the growth and progression of lumbar disc disease (LDD) [46]. The transcriptional activity of CASP-9 is intensified by polymorphism in the promoter region. This modulates the susceptibility to LDD [46]. Guo et al. studied the association between -712C/T (rs4645981) and CASP-9 -1263A/G (rs4645978) polymorphisms and discogenic LBP, finding that people with identified rs4645978 have a high probability of discogenic LBP. CASP-9 was found to be vital to regulating cell homeostasis through the cleavage of molecules concerned in apoptosis in mouse models, where the CASP-9 gene was made inoperative [47]. The apoptotic machinery within cells is engaged by numerous pro-apoptotic stimuli, leading to the generation of the apoptosome. The downstream CASP-9 cascade is then activated by the apoptosome with effector caspases, which leads to apoptosis [48]. Abnormal functioning apoptosomes are known to contribute to carcinogenesis, but may also play a role in various degenerative disorders [49,50]. Apoptosis inactivation is a hallmark of cancer, as it allows the survival of cells prone to genetic damage [51]. In contrast, apoptosis activation leads to cell reduction in the degenerated disc in LDD, particularly discogenic LBP. It is suggested by Guo et al. that the activity and/or frequency of CASP-9 could be greater in those who carry the -1263 GG genotype and that apoptosis of IVD cells may be abnormally enhanced in such individuals. Given that these disc cells possess and maintain a large extracellular matrix, the IVD being prone to degeneration with a reduced cell count is hardly surprising [52]. With advanced degeneration, radial tearing of the disc may occur [46].

#### 3.2.3. GCH1 (GTP Cyclohydrolase 1)

According to Tegeder et al., GTP hydrolase (GCH1) is a key modulator of neuropathic and inflammatory pain [53]. It is an enzyme that limits the rate of synthesis of BH4 (tetrahydrobiopterin). Downstream, BH4 affects production of serotonin, nitric oxide, and catecholamines. The amount of BH4 increases in primary sensory neurons after axonal injury due to the upregulation of GCH1. Dorsal root ganglia (DRGs) also see increased levels of BH4 after peripheral inflammation due to greater GCH1 activity. In rats, preventing new BH4 synthesis led to attenuation of inflammatory and neuropathic pain, and stopped nerve injury-related nitric oxide production in the DRP, whereas depositing BH4 intrathecally was found to aggravate pain. A haplotype of GCH1 found in 15.4% of the population was related to less pain after discectomy for persistent radicular low back pain in humans. Decreased pain sensitivity was shown in healthy test subjects homozygous for this haplotype. Leukocytes excited by forskolin in haplotype carriers saw less upregulated GCH1 than controls. In order to explore BH4’s possible implications in human pain, studies [53,54] have evaluated the possible relationship of certain pain phenotypes with polymorphisms in GCH1. Serious neurological issues and DOPA-responsive dystonia occur if BH4 is significantly decreased or nonexistent in humans, which takes place in uncommon instances of mutations—nonsense, missense, insertion, or deletion mutations in coding areas of GTP cyclohydrolase or sepiapterin reductase genes [55,56]. Due to the dependency of serotonin and dopamine neurotransmitter-synthesizing enzymes on BH4, inadequate amounts of BH4 lead to deficiencies of these transmitters and therefore, neurological conditions. This study found no neurological conditions in homozygotes for the pain-protective haplotype. It was consequently suggested that the pain-protective haplotype contains a variation in a regulatory site, leading to deterioration in GTP cyclohydrolase function or production. To further support this finding, the constitutive frequency of GTP cyclohydrolase and BH4 production was found to be the same between non-carriers and carriers of the pain-protective haplotype. These findings showed that changes in the amount of essential enzyme cofactor BH4 affect the sensitivity of the pain system. Further, the risk of developing continuous neuropathic pain and responses of healthy humans to noxious stimuli were both found to be affected by SNPs in the gene for the enzyme GTP cyclohydrolase. Since a decreased susceptibility to developing continuous pain is associated with the pain-protective haplotype in GCH1, there is potential for a treatment that might avoid the initial onset or development of chronic pain. This potential treatment could decrease surplus de novo synthesis of BH4 in the DRG, but not constitutive amounts of BH4, by leaving the recycling pathway untouched or by focusing solely on induction of GTP cyclohydrolase. Additionally, a factor that provides predictions into the severity and length of pain would also be a helpful device in analyzing a patient’s risk of chronic pain. The presence of BH4 in people suffering from inflammatory pain as well as peripheral neuropathy points to GCH1 upregulation as a result of overall injury to axons and thus, can predict the rate of chronic/postsurgical levels of pain [57,58].

#### 3.2.4. MMP 1,2,3 (Matrix Metallopeptidases)

Matrix metalloproteinases (MMPs) have an effect on the development of LBP due to their direct involvement in the deterioration of the extracellular matrix in the IVD. The -1607 promoter polymorphism, which is a SNP for guanine insertion/deletion (G/D) of the MMP1 gene, significantly affects transcription level and promoter activity. Song et al. [59] demonstrated an association between degenerative disc disease in southern Chinese subjects and the -1607 promoter polymorphism of MMP1. Genotypic association on the presence of the D allele as well as D allelic were significantly associated with DDD. Genotypic and allelic association were demonstrated by further age stratification in the group of subjects over 40 years old. The D allele was not associated with Schmorl’s nodes, disc bulges, or annular tears. Jacobsen et al. [60] have shown that inserting a SNP into the rs1799750 2G allele (promoter of MMP1) was associated with sciatica, LBP, and disability following lumbar disk herniation. These were measured by increased VAS scores, McGill pain questionnaire scores, and ODI scores. The presence of the rs1799750 2G allele is associated with the increase in in vitro MMP1 expression, but in clinical trials of patients with disk herniations, there were no differences in frequency of the allele when compared to pain-free controls. The MMP1 2G allele was not directly associated with disk degeneration in these patients. When compared to patients who were homozygous for the 2G allele, the patients who carried the 1G allele had less pain and were able to function better. The extracellular matrix within the IVD was prone to degradation where rs1799750 SNP was present because of increased MMP1 expression. Matrix degradation is thought to be principal in disk degeneration. As such, matrix metalloproteinase inhibitors have undergone clinical trials to try to treat neuropathic pain and multiple sclerosis. After nerve injury, the temporal and differential pattern of MMPs expression correlates with changes in concentrations of pro-inflammatory cytokines. This suggests that MMPs, besides being mediators for neuroinflammation, could also be directly associated with pain due to nerve damage. Blocking a single MMP with targeted treatments such as peptide inhibitors, monoclonal antibodies, and siRNAs can offer a better therapeutic approach while minimizing the adverse effects of broad-spectrum MMP inhibitors [61].

MMP2, matrix metalloproteinase-2, was demonstrated to contribute to the development of LDD. Amplified activity and expression of MMP2 were shown to be present in degenerative discs. There are reports of the polymorphism-1306C/T in the MMP-2 gene promoter influencing gene transcription and expression. LDD patients had a significantly greater prevalence of the MMP-2-1306CC genotype when compared to controls, as demonstrated by Don et al. [62]. CC-genotyped subjects had almost a three times greater risk for LDD development than did subjects who carried at least one T allele. On MR imaging, this genotype also corresponded with higher grade disc degeneration. Therefore, in young adults, the MMP-2-1306 C/T polymorphism may be a genetic risk factor linked to LDD susceptibility. Accelerated disc degeneration may result from increased expression of MMP-2 and subsequent tissue cleft formation and disc material resorption [62].

Matrix metalloproteinase-3 (MMP-3, stromelysin-1) has been implied in vertebral disc degeneration—specifically, 5a/6a polymorphism in the MMP3 promoter [63]. In elderly people, the 5A5A and 5A6A genotypes were associated with a notably larger number of degenerative IVDs and the degenerative scores were higher than in the 6A6A genotype. In younger people, there was no noted difference. This led to the conclusion that in the elderly, the 5A allele is a risk factor for accelerated lumbar disc changes. Omair et al. [64] found an association between improvement in pain at one year following lumbar fusion (*p* = 0.03) and with severe lumbar disc degeneration (*p* = 0.006) and MMP3 polymorphism rs72520913. Additionally, associations of severe degeneration with IL18RAP polymorphism rs1420100 and MMP3 polymorphism rs72520913 were observed in this study. The rs1420100 polymorphism was associated with more than one degenerated disc.

#### 3.2.5. FAAH (Fatty Acid Amide Hydrolase)

In a study by Ramesh et al., subjects who experienced both acute and low back pain at baseline demonstrated elevated levels of CNR2 mRNA; however, only subjects who went on to develop chronic LBP exhibited elevated levels of FAAH and TRPV1 mRNA [34]. Modest yet significantly elevated FAAH and TRPV1 expression were observed in those who developed cLBP compared to the acute LBP group, suggesting a possible genetic interaction that may increase vulnerability to chronic pain. Two SNPs within FAAH, rs932816 and rs4141964, were associated with increased pain scores on the McGill pain questionnaire among patients with LBP and accounted for ~5% variance in the pain ratings. The FAAH SNP rs932816 was significantly associated with the overall increased average pain and interference of pain among LBP patients [34]. Ethanolamine (anandamide, AEA) is an endogenous cannabinoid. Most of its pharmacological effects are via binding and activation of CB (1) and CB (2) cannabinoid receptors, in the periphery and the CNS [65]. Elevated levels of FAAH mRNA could lead to lower AEA levels and thus, dysregulation of normal pain processing [66]. In a study by Schlosburg et al., mice treated with FAAH inhibitors and FAAH knockout mice were unable to hydrolyze AEA along with other non-cannabinoid lipid signaling molecules. The animals with compromised FAAH persistently demonstrated phenotypes that were anti-inflammatory and antinociceptive, with efficacy comparable to direct-acting cannabinoid receptor agonists like THC [65]. However, a study performed on 74 patients with knee osteoarthritis found a lack of analgesic effect of a potent and selective FAAH1 inhibitor PF-04457845, despite decreasing activity of FAAH by >96% and increasing levels of the four endogenous substrates (fatty acid amides) [67]. The apparent disconnect between the animal models and human subjects warrants further investigation. 

Some relevant receptor-related gene studies that are not referenced in the text are listed in Table 2.

### 3.3. Cytokines and Associated Receptors

#### 3.3.1. IL18RAP (Interleukin 18 Receptor Accessory Protein); IL18R1 (Interleukin 18 Receptor 1); IL1A (Interleukin 1 Alpha)

Schistad et al. [69] reported that the C > T polymorphism rs1800587 in the interleukin-1α gene is associated with decreased pressure pain thresholds and increased pain intensity in patients with lumbar radicular pain. A pressure point threshold (PPT) was used to measure the pain severity for the gluteal muscles and VAS was used to measure the pain severity in the lower back and legs as the primary outcome. To determine the differences in genetic-makeup, a previously designed TaqMan assay was used for IL-1α rs1800587. By repeating analyses of variance with the different pain scores, the effect of the genotype was measured. After further analysis, the gene did have an effect on the scores in patients with symptomatic disk herniations. Patients who had CT/TT genotype had higher VAS pain scores for leg pain (*p* = 0.002) and lower PPT scores for the gluteus (*p* = 0.016 for both left and right side) compared to patients with the CC genotype during the 1-year follow-up. A study by Omair et al. [64] found that IL18RAP polymorphism rs1420100 was closely related to severe IVD degeneration in the lumbar segments (L4-L5 and L5-S1) and more than one degenerated IVD. Interestingly, SNPs rs917997 and rs1420106 from the same gene were linked to disequilibrium and with post treatment improvement in disability. The number of degenerated discs and degeneration severity associated with the rs1420100 SNP was confirmed by the study results of Videman et al. [70]. IL18RAP is important for IL18 signal transduction and ligand binding affinity, as it is a subunit of the IL18 receptor [71]. Secretion of interferon gamma (IFN-y) results from IL18R-induced activation of T cells and NK cells. The IFN activates macrophage cells to secrete Il-1 and TNF-alpha, leading to further production of cytokines and proteases and increased matrix degradation. The cells of herniated and degenerated discs secrete these proteases and cytokines [72,73,74]. This elucidates a link between inflammation and degeneration, and a viable pathway for back pain development. Significant associations with reduction in pain and improvement in disability were uncovered in association analysis of 5SNPs spanning the three genes (IL18RAP, IL18R1, IL1A).

#### 3.3.2. GDF5 (Growth Differentiation Factor 5)

In the Chinese Han population, Mu et al. [68] found that the GDF5 polymorphism (+104T/C; rs143383) was found to be associated with susceptibility to symptomatic lumbar disc herniation (LDH). Type II collagen in the nucleus pulposus of the disc may be an important component in susceptibility to symptomatic LDH [68]. The +104T/C variant increases the risk of developing musculoskeletal diseases and is the most prevalent SNP for GDF5. The SNP rs143383 is associated with osteoarthritis according to recent studies with replication studies, confirming this finding in different ethnic populaces [75,76]. In the Han Chinese cohort, the polymorphic T allele was less frequent in the control group than the case group. These results agreed with those of Williams et al., who observed SNP rs143383 association with lumbar disc degeneration in a cohort of Northern Europeans [77]. In this study, T allele and TT genotype were identified as predisposing to the risk of symptomatic lumbar disc herniation in both sexes.

#### 3.3.3. CCL2 (C-C Motif Chemokine Ligand 2)

Starkweather et al. [38] found chemokine (C-C motif) ligand 2 (CCL2) upregulation in the acute LBP group compared to no-pain controls. This gene has previously been shown in the oral surgery model of tissue injury and acute pain, with upregulation associated with pain intensity at three hours post op along with increased levels of proinflammatory cytokines [78]. 

Some relevant gene studies that are not referenced in the text are listed in Table 3.

### 3.4. Transcription Factors

#### 3.4.1. SOX5 (SRY-Box 5)

Loci tagged by rs7833174 (CCDC26/GSDMC), rs4384683 (DCC), and rs12310519 (SOX5) across the genome were significantly associated with chronic back pain (CBP), as demonstrated by Suri et al. [21]. Among the examined traits related to CBP, the lead SNP rs12310519 in SOX5 was closely linked with degeneration in the IVDs in the lumbar region [21,80]. SOX genes are transcription factors which are involved in all developments of the embryo, as they determine the outcomes for many cell types [21]. As SOX5 and SOX6 genes have some of the same functions, they are able to coordinate well together in order to efficiently undergo chondrogenesis [81]. When SOX5 was inactive, small defects in cartilage and skeleton formation in mice were noted. When both SOX5 and SOX6 were inactive, the mice had severe chondrodysplasia [82]. SOX5 and SOX6 are vital in the formation of IVDs, the spinal column, and notochord development [81,83]. If SOX5 and/or SOX6 are not active, mice with a range of spinal developmental issues and abnormalities are noted [84].

#### 3.4.2. CCDC26/GSDMC (CCDC26 Long Non-Coding RNA/Gasdermin C)

The lead SNP rs7833174 in CCDC26/GSDMC was known to mostly affect height and hip circumference in UKB (UK Biobank) [21]. It was also linked to radiographic hip osteoarthritis [85]. In a whole genome association study of Icelandic adults, all forms in CCFC26/GSDMC linked to CBP showed an interrelation with lumbar microdiscectomy for sciatica across phenotypes [86]. The effect direction was the same on other phenotypes as it was on CBP. For example, the T allele, which is associated with height increase, is also a prominent risk of osteoarthritis, CBP, and lumbar discectomy for sciatica [21]. Lumbar disc herniations bear some of the responsibility for causing forms of back pain [87]. Links between lumbar disc herniation and CBP can be clearly seen [88,89]. In the GSDM gene family, which is expressed in epithelial tissues, GASMC encodes for the protein Gasdermin C [21]. The role of GSDMC in lumbar disc herniation and sciatica is not known. In osteoarthritis-related cartilage and subchondral bone cartilage, it is usually linked to distinct methylation patterns [90,91]. After examining one variable genetic association for CBP at CCDC26/GSDMC across phenotypes, pleiotropy with radiographic hip OA at rs6470763 has been found [85]. These data suggest that there are links between variants at CCDC26/GSDMC and CBP [21].

#### 3.4.3. PNOC (Prepronociceptin)

PNOC is the gene which encodes prepronociceptin, a precursor to nociceptin. Nociceptin helps the opioid receptor-like receptor (OPRL1) bind to other molecules. The OPRL1 can modulate nociceptive behavior and movement by acting as a transmitter in the brain. Prepronociceptin appears to induce upregulation of cytokines and IL-10 decreases the expression of PNOC [92]. In the study by Starkweather et al. [38], upregulation of PNOC was associated with mechanical sensitivity of the painful region in the acute LBP group, suggesting a role in contributing to peripheral sensitization.

All genes related to transcription factors, neurotransmission and other unknown functions are shown in Table 4.

### 3.5. Pharmacogenomics in Management of cLBP

An estimated 70,000–100,000 people die each year from opioid overdoses from all around the world [93]. Nearly half of all opioid overdose deaths were from opioids that were prescribed to those individuals. According to the U.S. Drug Enforcement Administration (DEA), the amount of opioid overdoses has reached an epidemic level [94]. When patients with chronic pain are prescribed with opioid medications, there is a higher risk of the treatment having a poor outcome in the long run. Opioid therapy can have severe side effects such as misuse, overdose, hyperalgesia, and death. As improving a patient’s quality of life and functioning while avoiding adverse events is highly important, individualized therapies to treat chronic non-cancer pain are crucial [95]. In order for health care providers to be able to accurately diagnose and treat patients with chronic pain, they have to take into account variables like age, sex, ethnicity, lifestyle, comorbidities, and drugs that the patient may already be using. These factors combined with the contribution of genetics to the type of pain and efficacy and safety of drugs will ultimately impact the way that pharmacotherapy works.

The Human Pain Genetics Database (HPGDB) represents a large inventory of studies intended to summarize and reflect the association between genetic variations and different chronic pain conditions [96]. Interestingly, a specific phenotype category for which genetic associations were most frequently reported was analgesia. The Human Genome Research Project opened new opportunities for diagnosing diseases, developing drugs, and individualizing medicine. Personalized medicine in pain management has only been possible due to the advancements in research and technology, as well as the newly developed policies that empower patients [95]. Pharmacogenomics studies should help in discovering how an individual’s genome affects their response to pharmacotherapy. As such, it is a pathway to individualized treatment and can impact pharmacotherapy to maximize efficacy and minimize adverse reactions and polypharmacy.

**Figure 1:**
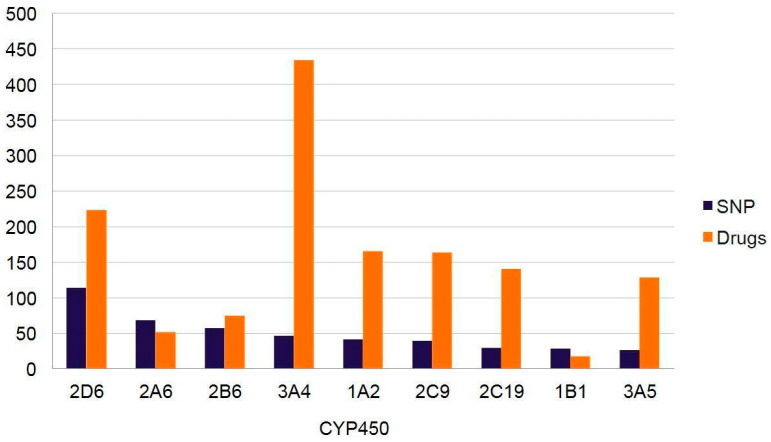
Number of known single nucleotide polymorphisms (SNPs) and drugs metabolized per cytochrome P450 (CYP) enzymes (modified from Preissner et al., 2013 [98]).

Cytochrome P450 (CYP450) is one of the most recognized superfamilies of enzymes responsible for inter-individual differences pertaining to drug effectiveness or adverse events profiles. Defined as membrane-associated proteins in the endoplasmic reticulum of cells, there are 57 genes identified coding for various CYP450 [97]. However, not all CYP types participate in drug metabolism. In the Caucasian population, a study associated four major CYP types (1A2, 2D6, 2C9, and 2C19) with 40.0% of drug metabolism [98] Moreover, in the same ethnic group, further analysis revealed 34 polymorphic alleles responsible for altered enzymatic activity. The authors also retrieved 199 non-synonymous SNPs with a prevalence of ≥1% in all genomes, irrespective of ethnicity (Figure 1). Prescribed analgesic drugs can have different effects on patients because of their genetic variations which contribute to the way they respond to the drugs, which is why pharmacogenomics plays a crucial role when dealing with pain management. Usually, the genetic variants in the CYP450 enzyme are what account for the different responses to drugs because of alteration to the protein structure and function. These variants are mostly known as single nucleotide polymorphisms [95]. The response to an analgesic medication therapy is highly dependent on prodrug metabolism, active component breakdown, and transport through cellular membranes [95].

Nonsteroidal anti-inflammatory drugs (NSAIDs) represent a commonly used class of drugs for the initial treatment of cLBP. To a large extent, the biotransformation of NSAIDs is governed by cytochrome P450 isoforms, in particular by CYP2C9 [99]. Adjusted to CYP2C9 activity score, the Clinical Pharmacogenetics Implementation Consortium (CPIC) retrieved three distinct CYP2C9 phenotypes: poor metabolizers (PM), intermediate metabolizers (IM), and normal metabolizers (NM) [100]. PM and IM variants are linked with decreased metabolic clearance potential, which results in a prolonged plasma elimination half-life of NSAIDs. In addition, CYP2C9*3 was associated with decreased celecoxib, meloxicam, as well as S (+) and R (−) ibuprofen metabolism. The same genotype also rendered meloxicam with enhanced pharmacodynamic effects (increased inhibition of thromboxane B_2_ formation). Gastroduodenal bleeding, a serious NSAID-related adverse event, has been found highly probable in CYP2C9*1/*3 and CYP2C9*1/*2 heterozygotes [101] A later study recognized the CYP2C9 359Leu (CYP2C9*3) allele as a risk factor for acute upper gastrointestinal bleeding in patients taking NSAIDs other than aspirin.

In chronic pain management, the CYP450 polymorphisms are also relevant in the metabolism of opioid drugs like codeine, tramadol, hydrocodone, and oxycodone. Their use, while common in pain management, can lead to unpredictable and sometimes dangerous consequences. Hepatic cytochrome P450 2D6 (CYP2D6) is pivotal for bioactivating codeine into morphine, and tramadol into *O*-desmethyltramadol. The clinical significance from CYP2D6 polymorphism would render an individual susceptible to variable outcomes to efficacy and safety profiles of codeine. Accordingly, CPIC guidelines have classified different patient phenotypes with respect to the CYP2D6 activity score [102]. The authors identified four such profiles: PM, IM, extensive metabolizer (EM (normal morphine formation)), and ultrarapid metabolizer (UM). PM variants can significantly reduce the activity of drug metabolism and lead to insufficient pain relief and lower drug clearance, requiring a reduction in the drug dose to avoid undesired adverse effects. For this phenotype, it is recommended to consider drugs such as morphine or use of a non-opioid [102]. Reduced codeine metabolism is also seen with IM, although not as pronounced as with PM, and therapy protocols in such phenotypes advocate for a trial of codeine as a first-line opioid analgesic. If no response is identified, second tier drugs would include morphine, use of a non-opioid, or tramadol. Finally, UM, as the least prevalent and most extensive metabolic-capable variant, has been described as being high risk for morphine toxicity; codeine should be avoided, and clinicians should instead opt for morphine or a non-opioid. Put into perspective, a retrospective cohort of 224 patients with CLBP treated with oxycodone or codeine were analyzed with respect to their CYP26D genotype [103]. There were statistically significant findings in regard to therapeutic failures at the haplotype (CYP2D6*6 (PM) and CYP2D6*9 (IM)) as well as diplotype level (CYP2D6 *1/*11 (EM), *4/*6 (PM), *41/*2N (UM)) with chronic opioid treatment (*p* < 0.05). Moreover, CYP2D6*2N patients exhibited increased risks of side effects. A prospective cohort study with 76 chronic pain patients receiving codeine or tramadol was conducted to assess the prevalence of CYP2D6 genotype among the cohort [104]. The authors analyzed the nine most common variants (CYP2D6 *2–6, *9, *10, *14, and *17), as well as those without polymorphic alleles (CYP2D6*1, wild type (wt)). The most common genotypes per se as well as adverse effects among such variants were identified, thus paving the path for a more personalized therapy.

Moreover, the link between hydromorphone and OPRM1 A118A genotypes (homozygous (AA) vs. heterozygous (AG)) was explored in 158 women receiving hydrocodone/acetaminophen postoperatively following Cesarean section [105]. Patients homozygous for the A118A allele had statistically significant pain relief associated with both the total dose of hydrocodone and serum hydromorphone level, while adverse events more commonly occurred in the heterozygous group. The CYP450 enzyme family is not the only one that affects pain management. A study with 231 opioid-naïve patients revealed that those with the COMT G472A-AA genotype (rs4680) and KCNJ6 A1032G-A allele (rs2070995) required higher dosing. When a higher pain intensity was present, they responded differently to opioid titration with higher pain intensity, thereby requiring higher dosing [106] The single-nucleotide polymorphisms in genes closely related to pain transmission and the metabolism of opioids may cause patients with cLBP to possibly be predisposed to excessive sensitivity and variation in the effects of opioid analgesics.

The management of different chronic pain conditions includes adjunct drugs such as antidepressants, muscle relaxants, and anticonvulsants. The three major CYP enzymes implicated in the metabolism of antidepressants are CYP1A2, CYP2D6, and CYP2C19; however, other enzymes are also involved as evidenced by the metabolism of amitriptyline (1A2, 2C9, 2C19, 2D6, 3A), bupropion (2B6), imipramine (1A2, 2C19, 2D6, 3A), venlafaxine (2D6, 3A), etc. [107,108,109]. Nevertheless, CYP2D6 isoenzyme has been most extensively studied in regards to antidepressant metabolism. Indeed, CYP2D6 phenotypes predispose to large differences in plasma drug concentrations and variable rates of adverse events [107,110]. The Royal Dutch Association of the Advancement of Pharmacy developed pharmacogenetics-based guidelines for a number of drugs including venlafaxine [111]. For PM and IM phenotypes, the recommendations were to select an alternate drug (e.g., citalopram, sertraline) or adjust dose and monitor O-desmethylvenlafaxine, a venlafaxine metabolite. In contrast, for the UM phenotype, it is recommended to titrate to a maximum of 150% of the normal dose or opt for one of the abovementioned alternative drugs. Of note, the efficacy and safety of venlafaxine has been associated with SNPs rs2032582 (G2677T) and rs1045642 (C3435T) within the ABCB1 gene that codes for membrane-bound P-glycoprotein (P-gp) [112,113,114] Genetic polymorphism for serotonin transporter 5-HT (5-HTTLPR), characterized by short (s) and long (l) variants, has been associated with the efficacy of another antidepressant, citalopram [115] Among l/l 5-HTTLPR homozygotes, citalopram significantly reduces pain-related responses in the cerebellum and in parts of the cerebral cortex, while the relationship between the 5-HTTLPR genotype and pain-related brain response was shown to be a good predictor of pain alleviating properties of citalopram. Duloxetine, a serotonin and norepinephrine reuptake inhibitor, is another antidepressant drug whose metabolism is amenable to certain CYP (primarily CYP1A2, but also CYP2D6 and CYP2C9) enzymes. For this reason, one should expect potentially toxic plasma levels of duloxetine in the case of concomitant administration of a strong CYP1A2 inhibitor [95].

The pharmacokinetic properties of a commonly used muscle relaxant, cyclobenzaprine, were the subject of investigation in four clinical studies [116]. It was shown that steady-state plasma concentrations of this drug were two-fold higher in the elderly population and those individuals with hepatic insufficiency, necessitating dose reduction in such patient groups. In addition, there is preclinical evidence that rendered the therapeutic plasma levels of cyclobenzaprine accountable for the initiation of serotonin syndrome, a potentially fatal condition characterized by altered mental status and autonomic instability [117].

The activity of some of the aforementioned CYP enzymes, such as CYP3A4, CYP2C9, and CYP2C19, may be affected by different anticonvulsants in a stimulating (phenytoin, carbamazepine) or inhibitory (oxcarbazepine, valproic acid) fashion, thus creating an environment for adverse drug reactions and drug–drug interactions [118]. In contrast, gabapentinoids (gabapentin, pregabalin) are neither activators/inhibitors of the cytochrome P450 system nor subject to hepatic metabolism [119,120], but are instead excreted in urine. This process is under the influence of organic cation transporters OCTN1 and OCT2 coded by SLC22A4 and SLC22A2 genes, respectively [121,122,123]. However, the genotype of an individual (e.g., OCTN1 polymorphism) was found to have a negligible role in gabapentin clearance and was much more affected by the renal function and absorption process [123].

#### 3.5.1. Drug–Drug Interactions

**Figure 2:**
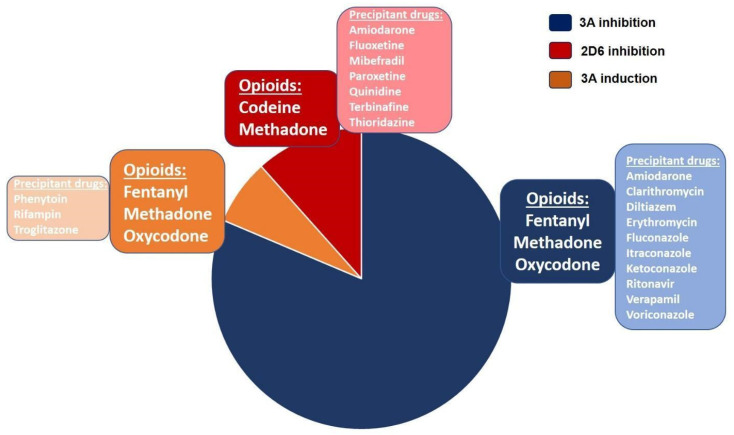
Most common CYP450-related pharmacokinetic drug–drug interactions between opioids and precipitant drugs (modified with permission from Pergolizzi et al. [125]).

An additional shortfall of current cLBP management is the unfortunate circumstance of polypharmacy use, and with it, drug–drug interactions (DDIs), where the toxicity and/or efficacy of one or all drugs is altered. If the metabolism of the used drugs (several opioids) goes through the cytochrome P450 (CYP450) pathways, the patient is possibly exposed to dangerous DDIs. The overall prevalence of DDIs among cLBP is 27% [124]. A large retrospective cohort analysis was conducted to assess for pharmacokinetic drug–drug interactions (pDDI) in 57,752 chronic non-cancer pain patients taking opioids [125] The authors matched the 9 most commonly prescribed opioids against 19 precipitant drugs capable of inducing CYP450-dependent metabolic effects changes, and sought for those pDDIs with a potential to induce adverse drug reactions (i.e., PDDI-major). In a decreasing order of frequency, the most prevalent pDDIs were caused from 3A inhibition, followed by 2D6 inhibition and 3A induction, while the leading precipitant drugs included fluconazole, followed by diltiazem, clarithromycin, and verapamil. The summary of the most common, CYP450-related, prescribed opioids and precipitant drugs in chronic non-cancer pain patients is shown in Figure 2. About 5.7% of the cohort was found to have been exposed to potential PDDI-major, and these had significantly higher healthcare costs vs. patients without a drug–drug interaction.

#### 3.5.2. Drug–Drug–Gene Interactions

Knowing a patient’s genotyping as part of an overall clinical practice may lead to better outcomes. Pharmacogenomics and therapeutic drug monitoring can potentially minimize adverse events, while maximizing efficacy. The incidence of adverse events depends on a number of variables, including sex, age, comorbidities, genetic variations, etc. Indeed, a recent retrospective analysis in patients with known genetic polymorphisms in major drug metabolizing enzymes (CYP2D6, CYP2C9, and CYP2C19) revealed that drug–gene (DG) and drug–drug–gene (DDG) interactions accounted for 14.7% and 19.2% of adverse events [126]. DDG interactions have been classified into three categories: inhibitory, induction, and phenoconversion interactions [127]. Inhibitory and induction interactions assume altered metabolic and pharmacokinetic properties of the target drug, which can be influenced by the presence of another drug, genetic mutations of relevant enzymes, or their combination. Phenoconversion is related to opposing effects between the effect of the interacting drug and the genotype, which practically would make genetically susceptible individuals normalized by adding medications having opposite effects on metabolism. Moreover, drug–drug–gene interactions have also been proposed to influence drug transporters (i.e., drug–drug–transporters genes interaction), and subsequently, drug pharmacokinetics, in a similar fashion as with drug metabolizing enzymes. Storelli et al. managed to render physiologically based pharmacokinetic (PBPK) modeling appropriate to predict the influence of CYP2D6 genetic polymorphisms on DDIs [128]. The clinical significance behind PBPK simulations lies in personalized medicine—to help identify individuals susceptible to higher risk of DDIs and deliver a genotype-specific drug dose. Incorporating genetic analysis into clinical studies can help predict responses to different treatment options by identifying clinical and genetic factors. When the treating physician knows how a patient might respond to a given therapy, this can help them guide which therapies they might prescribe. This form of personalized medicine with incorporated biomarkers helps to drastically improve the effectiveness of current and future strategies in pain management [129].

## 4. Conclusions

Treatment of chronic low back pain (cLBP) should be multimodal. We hope that with future research, it will be possible to use genetic markers for identifying patients at risk for developing cLBP early. Additionally, genotyping may assist in directing treatment, predicting lack of efficacy with any particular approach, and facilitating decisions between conservative approaches or early escalations to more radical approaches such as surgery. Examining genetic markers could provide objective data for physicians treating cLBP, instead of relying upon more subjective measures such as numeric pain rating scales. Making a “pain profile” of a patient, which would include genetic markers, while being costly at present, could lead to minimizing healthcare costs in the future, by getting the patients the personalized treatment that they require early, and by minimizing inefficacious approaches. In order to deliver a more personalized therapy, further research is warranted to explore how an individual’s genotype affects their responses to therapy. In particular, the focus should be on genetic polymorphisms in CYP450 and other enzymes crucial for affecting the metabolic profile of target drugs. Implementation of gene-focused pharmacotherapy has the potential to deliver select, more efficacious drugs and avoid unnecessary, polypharmacy-related adverse events in many painful conditions, including chronic low back pain.

Our review provides some targets for future research into pharmacogenomics. It is our hope that by obtaining additional knowledge regarding polymorphisms in these genes and their relationship to pharmacotherapy response, we will help guide future therapies, reduce overall healthcare costs, and prevent perpetuation of the opioid epidemic, among other benefits.

## Figures and Tables

**Table 1:** Receptor-related genes.

Gene	Function/Pathway	Condition(s)	Citation	Number of Subjects/Geographic Region
DCC	Receptor for Netrin-1, as an axonal guidance molecule	LBP	Suri et al., 2018 [21]	*n* = 168,000
ESR1	Other/Estrogen receptor 1	LBP	Roh et al., 2013 [29]	*n* = 192, South Korea
ADRB2	Neurotransmission/beta-2 adrenergic receptor	TMD/LBP/Fibromyalgia LBP comorbid with neck pain	Diatchenko et al., 2006 [36]/Skouen et al. [35]/Vargas-Alarcon et al., 2009 [37]	*n* = 1004; Western Australian Pregnancy (Raine) Cohort
CNR2	Peripheral cannabinoid receptor; nociceptive transmission, inflammatory response, bone homeostasis	LBP/mechanical allodynia, neuroinflammation in CRPS1/Joint pain	Starkweather et al., 2017 [38]; Ramesh et al., 2018 [34]/Xu et al., 2016 [39]	*n* = 62 USA; *n* = 84 USA/animal model/animal model
OPRM1	Neurotransmission/Mu opioid receptor	LBP	Hasvik et al., 2014 [10], Omair et al. have not replicated the above (2015) [40]	*n* = 118 Caucasians, Norway

Abbreviations: TMD, temporomandibular disorder; LBP, low back pain; CRPS1, complex regional pain syndrome 1; DCC, deleted in colorectal carcinoma.

**Table 2:** Enzyme-related genes.

Gene	Function/Pathway	Condition(s)	Citation	Number of Subjects/Geographic Region
FAAH	hydrolyzes many primary and secondary fatty acid amides, including anandamide and oleamide as neuromodulators	cLBP	Ramesh et al., 2018 [34]	*n* = 84, USA
COMT	Neurotransmission/Catechol-O-methyltransferase	LBP	Rut et al., 2014 [45], Jacobsen et al., 2012 [44], Omair et al., 2013, 2015 [40,64]	*n* = 176, Poland/*n* = 258, Norway/*n* = 93, Norway (West Eur), *n* = 371, Europe
GCH	Guanosine triphosphate cyclohydrolase	LBP	Tegeder et al., 2006 [53]	Animal studies
MMP1	Protein degradation/matrix metalloproteinase	LBP	Song et al., 2008 [59] Jacobsen et al., 2013 [60]	*n* = 691, southern China; *n* = 260, Norway Eur White
MMP2	Protein degradation	LBP	Dong et al., 2007 [62]	*n* = 162, China
MMP3	Protein degradation	LBP	Takahashi et al., 2001 [63]	*n* = 103, Japan
CASP9	Apoptosis-mediating caspase	LBP	Guo et al., 2011 [46], Mu et al., 2013 [68]	*n =* 154/216 controls in China; *n* = 305/587 controls Chinese soldiers

Abbreviations: LBP, low back pain; cLBP, chronic low back pain.

**Table 3:** Genes related to cytokines and their associated receptors.

Gene	Function/Pathway	Condition(s)	Citation	Number of Subjects/Geographic Region
CCL2	Chemotactic factor for monocytes and basophils	LBP	Starkweather et al., 2017 [38]	*n =* 62, USA
IL18R1 IL18RAP IL1A	Immune response/Interleukin receptors	LBP	Omair et al., 2013 [64] Schistad et al., 2014 [69]	*n* = 93, Norway; *n* = 121, Norway
GDF5	Part of TGF-beta family, Cellular growth/Skeletal tissue differentiation	LBP	Mu et al., 2013 [79]	*n* = 305/587 controls Chinese soldiers

Abbreviations: LBP, low back pain.

**Table 4:** Genes related to transcription factors, neurotransmission, and other unknown functions.

Gene	Function/Pathway	Condition(s)	Citation	Number of Subjects/Geographic Location
SOX5	Transcription factor, embryonic development	LBP	Suri et al., 2018 [21]	*n* = 168,000; worldwide
CCDC26/GSDMC	Non-coding/Codes gasdermin C; the N-terminal moiety promotes pyroptosis with unknown physiologic significance	LBP	Suri et al., 2018 [21]	*n* = 168,000; worldwide
PNOC	Codes prepronociceptin; nociceptin is a ligand of the opioid receptor-like receptor OPRL1; may modulate nociceptive and locomotor behavior	LBP	Starkweather et al., 2016 [38]	*n* = 62, CT USA

Abbreviations: LBP, low back pain.

## References

[1] Manchikanti L., Singh V., Falco F.J., Benyamin R.M., Hirsch J.A. (2014). Epidemiology of low back pain in adults. Neuromodulation.

[2] Shmagel A., Foley R., Ibrahim H. (2016). Epidemiology of Chronic Low Back Pain in US Adults: Data from the 2009-2010 National Health and Nutrition Examination Survey. Arthritis Care Res..

[3] Polderman T.J., Benyamin B., de Leeuw C.A., Sullivan P.F., Van Bochoven A., Visscher P.M., Posthuma D. (2015). Meta-analysis of the heritability of human traits based on fifty years of twin studies. Nat. Genet..

[4] Ferreira P.H., Beckenkamp P., Maher C.G., Hopper J.L., Ferreira M.L. (2013). Nature or nurture in low back pain? Results of a systematic review of studies based on twin samples. Eur. J. Pain.

[5] Battie M.C., Videman T., Levalahti E., Gill K., Kaprio J. (2007). Heritability of low back pain and the role of disc degeneration. Pain.

[6] Rodriguez-Raecke R., Niemeier A., Ihle K., Ruether W., May A. (2013). Structural brain changes in chronic pain reflect probably neither damage nor atrophy. PLoS ONE.

[7] Baliki M.N., Petre B., Torbey S., Herrmann K.M., Huang L., Schnitzer T.J., Fields H.L., Apkarian A.V. (2012). Corticostriatal functional connectivity predicts transition to chronic back pain. Nat. Neurosci..

[8] Seminowicz D.A., Wideman T.H., Naso L., Hatami-Khoroushahi Z., Fallatah S., Ware M.A., Jarzem P., Bushnell M.C., Shir Y., Ouellet J.A. (2011). Effective treatment of chronic low back pain in humans reverses abnormal brain anatomy and function. J. Neurosci..

[9] Zorina-Lichtenwalter K., Meloto C.B., Khoury S., Diatchenko L. (2016). Genetic predictors of human chronic pain conditions. Neuroscience.

[10] Hasvik E., Iordanova Schistad E., Grovle L., Julsrud Haugen A., Roe C., Gjerstad J. (2014). Subjective health complaints in patients with lumbar radicular pain and disc herniation are associated with a sex—OPRM1 A118G polymorphism interaction: A prospective 1-year observational study. BMC Musculoskelet. Disord..

[11] Grovle L., Haugen A.J., Ihlebaek C.M., Keller A., Natvig B., Brox J.I., Grotle M. (2011). Comorbid subjective health complaints in patients with sciatica: A prospective study including comparison with the general population. J. Psychosom. Res..

[12] Zubieta J.K., Dannals R.F., Frost J.J. (1999). Gender and age influences on human brain mu-opioid receptor binding measured by PET. Am. J. Psychiatry.

[13] Ray R., Ruparel K., Newberg A., Wileyto E.P., Loughead J.W., Divgi C., Blendy J.A., Logan J., Zubieta J.-K., Lerman C. (2011). Human Mu Opioid Receptor (OPRM1 A118G) polymorphism is associated with brain mu-opioid receptor binding potential in smokers. Proc. Natl. Acad. Sci. USA.

[14] Matsunaga M., Isowa T., Murakami H., Kasugai K., Yoneda M., Kaneko H., Ohira H. (2009). Association of polymorphism in the human mu-opioid receptor OPRM1 gene with proinflammatory cytokine levels and health perception. Brain Behav. Immun..

[15] Way B.M., Taylor S.E., Eisenberger N.I. (2009). Variation in the mu-opioid receptor gene (OPRM1) is associated with dispositional and neural sensitivity to social rejection. Proc. Natl. Acad. Sci. USA.

[16] Milner T.A., Burstein S.R., Marrone G.F., Khalid S., Gonzalez A.D., Williams T.J., Schierberl K.C., Torres-Reveron A., Gonzales K.L., McEwen B.S. (2013). Stress differentially alters mu opioid receptor density and trafficking in parvalbumin-containing interneurons in the female and male rat hippocampus. Synapse.

[17] Nicholl B.I., Holliday K.L., Macfarlane G.J., Thomson W., Davies K.A., O’Neill T., Bartfai G., Boonen S., Casanueva F.F., Finn J.D. (2011). Association of HTR2A polymorphisms with chronic widespread pain and the extent of musculoskeletal pain: Results from two population-based cohorts. Arthritis Rheum..

[18] Yildiz S.H., Ulasli A.M., Ozdemir Erdogan M., Dikici Ö., Terzi E.S.A., Dündar Ü., Solak M. (2017). Assessment of Pain Sensitivity in Patients with Chronic Low Back Pain and Association with HTR2A Gene Polymorphism. Arch. Rheumatol..

[19] Kato K., Kikuchi S., Konno S., Sekiguchi M. (2008). Participation of 5-hydroxytryptamine in pain-related behavior induced by nucleus pulposus applied on the nerve root in rats. Spine.

[20] Kanayama M., Hashimoto T., Shigenobu K., Yamane S. (2003). Efficacy of serotonin receptor blocker for symptomatic lumbar disc herniation. Clin. Orthop. Relat. Res..

[21] Suri P., Palmer M.R., Tsepilov Y.A., Freidin M.B., Boer C.G., Yau M.S., Evans D.S., Gelemanović A., Bartz T.M., Nethander M. (2018). Genome-wide meta-analysis of 158,000 individuals of European ancestry identifies three loci associated with chronic back pain. PLoS Genet..

[22] Finci L., Zhang Y., Meijers R., Wang J.H. (2015). Signaling mechanism of the netrin-1 receptor DCC in axon guidance. Prog. Biophys. Mol. Biol..

[23] Wu C.H., Yuan X.C., Gao F., Li H.-P., Cao J., Liu Y.-S., Yu W., Tian B., Meng X.-F., Shi J. (2016). Netrin-1 Contributes to Myelinated Afferent Fiber Sprouting and Neuropathic Pain. Mol. Neurobiol..

[24] Dun X.P., Parkinson D.B. (2017). Role of Netrin-1 Signaling in Nerve Regeneration. Int. J. Mol. Sci..

[25] Bu G., Hou S., Ren D., Wu Y., Shang W., Huang W. (2012). Increased expression of netrin-1 and its deleted in colorectal cancer receptor in human diseased lumbar intervertebral disc compared with autopsy control. Spine.

[26] Freemont A.J., Peacock T.E., Goupille P., Hoyland J.A., O’Brien J., Jayson M.I. (1997). Nerve ingrowth into diseased intervertebral disc in chronic back pain. Lancet.

[27] Pinheiro M.B., Ferreira M.L., Refshauge K., Colodro-Conde L., Carrillo E., Hopper J.L., Ordoñana J.R., Ferreira P.H. (2015). Genetics and the environment affect the relationship between depression and low back pain: A co-twin control study of Spanish twins. Pain.

[28] Okbay A., Baselmans B.M., De Neve J.E., Turley P., Nivard M.G., Fontana M.A., Meddens S.F.W., Linnér R.K., Rietveld C.A., LifeLines Cohort Study (2016). Genetic variants associated with subjective well-being, depressive symptoms, and neuroticism identified through genome-wide analyses. Nat. Genet..

[29] Roh H.L., Lee J.S., Suh K.T., Kim J.I., Lee H.S., Goh T.S., Park S.H. (2013). Association between estrogen receptor gene polymorphism and back pain intensity in female patients with degenerative lumbar spondylolisthesis. J. Spinal Disord. Tech..

[30] Lindberg M.K., Alatalo S.L., Halleen J.M., Mohan S., Gustafsson J.A., Ohlsson C. (2001). Estrogen receptor specificity in the regulation of the skeleton in female mice. J. Endocrinol..

[31] Vidal O., Lindberg M.K., Hollberg K., Baylink D.J., Andersson G., Lubahn D.B., Mohan S., Gustafsson J.-Å., Ohlsson C. (2000). Estrogen receptor specificity in the regulation of skeletal growth and maturation in male mice. Proc. Natl. Acad. Sci. USA.

[32] Ushiyama T., Ueyama H., Inoue K., Nishioka J., Ohkubo I., Hukuda S. (1998). Estrogen receptor gene polymorphism and generalized osteoarthritis. J. Rheumatol..

[33] Racz I., Nadal X., Alferink J., Banos J.E., Rehnelt J., Martin M., Pintado B., Gutierrez-Adan A., Sanguino E., Manzanares J. (2008). Crucial role of CB(2) cannabinoid receptor in the regulation of central immune responses during neuropathic pain. J. Neurosci..

[34] Ramesh D., D’Agata A., Starkweather A.R., Young E.E. (2018). Contribution of Endocannabinoid Gene Expression and Genotype on Low Back Pain Susceptibility and Chronicity. Clin. J. Pain.

[35] Skouen J.S., Smith A.J., Warrington N.M., O’Sullivan P., McKenzie L., Pennell C.E., Straker L. (2012). Genetic variation in the beta-2 adrenergic receptor is associated with chronic musculoskeletal complaints in adolescents. Eur. J. Pain.

[36] Diatchenko L., Anderson A.D., Slade G.D., Fillingim R.B., Shabalina S.A., Higgins T.J., Sama S., Belfer I., Goldman D., Max M.B. (2006). Three major haplotypes of the beta2 adrenergic receptor define psychological profile, blood pressure, and the risk for development of a common musculoskeletal pain disorder. Am. J. Med. Genet. B Neuropsychiatr. Genet..

[37] Vargas-Alarcon G., Fragoso J.M., Cruz-Robles D., Vargas A., Martinez A., Lao J.I., Garcia-Fructuoso F., Vallejo M., Martínez-Lavín M. (2009). Association of adrenergic receptor gene polymorphisms with different fibromyalgia syndrome domains. Arthritis Rheum..

[38] Starkweather A.R., Ramesh D., Lyon D.E., Siangphoe U., Deng X., Sturgill J., Heineman A., Elswick R.K., Dorsey S.G., Greenspan J. (2016). Acute Low Back Pain: Differential Somatosensory Function and Gene Expression Compared with Healthy No-Pain Controls. Clin. J. Pain.

[39] Xu J., Tang Y., Xie M., Bie B., Wu J., Yang H., Foss J., Yang B., Rosenquist R.W., Naguib M. (2016). Activation of cannabinoid receptor 2 attenuates mechanical allodynia and neuroinflammatory responses in a chronic post-ischemic pain model of complex regional pain syndrome type I in rats. Eur. J. Neurosci..

[40] Omair A., Mannion A.F., Holden M., Fairbank J., Lie B.A., Hägg O., Fritzell P., Brox J.I. (2015). Catechol-O-methyltransferase (COMT) gene polymorphisms are associated with baseline disability but not long-term treatment outcome in patients with chronic low back pain. Eur. Spine J..

[41] Nackley A.G., Tan K.S., Fecho K., Flood P., Diatchenko L., Maixner W. (2007). Catechol-O-methyltransferase inhibition increases pain sensitivity through activation of both beta2- and beta3-adrenergic receptors. Pain.

[42] Jacobsen L.M., Eriksen G.S., Pedersen L.M., Gjerstad J. (2010). Catechol-O-methyltransferase (COMT) inhibition reduces spinal nociceptive activity. Neurosci. Lett..

[43] Zubieta J.K., Heitzeg M.M., Smith Y.R., Bueller J.A., Xu K., Koeppe R.A., Stohler C.S., Goldman D. (2003). COMT val158met genotype affects mu-opioid neurotransmitter responses to a pain stressor. Science.

[44] Jacobsen L.M., Schistad E.I., Storesund A., Pedersen L., Rygh L., Røe C., Gjerstad J. (2012). The COMT rs4680 Met allele contributes to long-lasting low back pain, sciatica and disability after lumbar disc herniation. Eur. J. Pain.

[45] Rut M., Machoy-Mokrzynska A., Reclawowicz D., Słoniewski P., Kurzawski M., Droździk M., Safranow K., Morawska M., Białecka M. (2014). Influence of variation in the catechol-*O*-methyltransferase gene on the clinical outcome after lumbar spine surgery for one-level symptomatic disc disease: A report on 176 cases. Acta Neurochir..

[46] Guo T.M., Liu M., Zhang Y.G., Guo W.T., Wu S.X. (2011). Association between Caspase-9 promoter region polymorphisms and discogenic low back pain. Connect. Tissue Res..

[47] Kuida K., Haydar T.F., Kuan C.Y., Gu Y., Taya C., Karasuyama H., Su M.S.-S., Rakic P., Flavell R.A. (1998). Reduced apoptosis and cytochrome c-mediated caspase activation in mice lacking caspase 9. Cell.

[48] Srinivasula S.M., Ahmad M., Fernandes-Alnemri T., Alnemri E.S. (1998). Autoactivation of procaspase-9 by Apaf-1-mediated oligomerization. Mol. Cell..

[49] Shivapurkar N., Reddy J., Chaudhary P.M., Gazdar A.F. (2003). Apoptosis and lung cancer: A review. J. Cell Biochem..

[50] Sang T.K., Li C., Liu W., Rodriguez A., Abrams J.M., Zipursky S.L., Jackson G.R. (2005). Inactivation of Drosophila Apaf-1 related killer suppresses formation of polyglutamine aggregates and blocks polyglutamine pathogenesis. Hum. Mol. Genet..

[51] Hanahan D., Weinberg R.A. (2000). The hallmarks of cancer. Cell.

[52] Zhao C.Q., Jiang L.S., Dai L.Y. (2006). Programmed cell death in intervertebral disc degeneration. Apoptosis.

[53] Tegeder I., Costigan M., Griffin R.S., Abele A., Belfer I., Schmidt H., Ehnert C., Nejim J., Marian C., Scholz J. (2006). GTP cyclohydrolase and tetrahydrobiopterin regulate pain sensitivity and persistence. Nat. Med..

[54] Lotsch J., Klepstad P., Doehring A., Dale O. (2010). A GTP cyclohydrolase 1 genetic variant delays cancer pain. Pain.

[55] Ichinose H., Ohye T., Takahashi E., Seki N., Hori T.-A., Segawa M., Nomura Y., Endo K., Tanaka H., Tsuji S. (1994). Hereditary progressive dystonia with marked diurnal fluctuation caused by mutations in the GTP cyclohydrolase I gene. Nat. Genet..

[56] Bonafe L., Thony B., Penzien J.M., Czarnecki B., Blau N. (2001). Mutations in the sepiapterin reductase gene cause a novel tetrahydrobiopterin-dependent monoamine-neurotransmitter deficiency without hyperphenylalaninemia. Am. J. Hum. Genet..

[57] Bisgaard T., Klarskov B., Rosenberg J., Kehlet H. (2001). Characteristics and prediction of early pain after laparoscopic cholecystectomy. Pain.

[58] Bisgaard T., Rosenberg J., Kehlet H. (2005). From acute to chronic pain after laparoscopic cholecystectomy: A prospective follow-up analysis. Scand. J. Gastroenterol..

[59] Song Y.Q., Ho D.W., Karppinen J., Kao P.Y.P., Fan B.J., Luk K.D.K., Yip S.P., Leong J.C.Y., Cheah K.S.E., Sham P.C. (2008). Association between promoter -1607 polymorphism of MMP1 and lumbar disc disease in Southern Chinese. BMC Med. Genet..

[60] Jacobsen L.M., Schistad E.I., Storesund A., Pedersen L.M., Espeland A., Rygh L.J., Røe C., Gjerstad J. (2013). The MMP1 rs1799750 2G allele is associated with increased low back pain, sciatica, and disability after lumbar disk herniation. Clin. J. Pain.

[61] Dev R., Srivastava P.K., Iyer J.P., Dastidar S.G., Ray A. (2010). Therapeutic potential of matrix metalloprotease inhibitors in neuropathic pain. Expert Opin. Investig. Drugs.

[62] Dong D.M., Yao M., Liu B., Sun C.Y., Jiang Y.Q., Wang Y.S. (2007). Association between the -1306C/T polymorphism of matrix metalloproteinase-2 gene and lumbar disc disease in Chinese young adults. Eur. Spine J..

[63] Takahashi M., Haro H., Wakabayashi Y., Kawa-uchi T., Komori H., Shinomiya K. (2001). The association of degeneration of the intervertebral disc with 5a/6a polymorphism in the promoter of the human matrix metalloproteinase-3 gene. J. Bone Jt. Surg. Br..

[64] Omair A., Holden M., Lie B.A., Reikeras O., Brox J.I. (2013). Treatment outcome of chronic low back pain and radiographic lumbar disc degeneration are associated with inflammatory and matrix degrading gene variants: A prospective genetic association study. BMC Musculoskelet. Disord..

[65] Schlosburg J.E., Kinsey S.G., Lichtman A.H. (2009). Targeting fatty acid amide hydrolase (FAAH) to treat pain and inflammation. AAPS J..

[66] Piomelli D., Sasso O. (2014). Peripheral gating of pain signals by endogenous lipid mediators. Nat. Neurosci..

[67] Huggins J.P., Smart T.S., Langman S., Taylor L., Young T. (2012). An efficient randomised, placebo-controlled clinical trial with the irreversible fatty acid amide hydrolase-1 inhibitor PF-04457845, which modulates endocannabinoids but fails to induce effective analgesia in patients with pain due to osteoarthritis of the knee. Pain.

[68] Mu J., Ge W., Zuo X., Chen Y., Huang C. (2014). A SNP in the 5′UTR of GDF5 is associated with susceptibility to symptomatic lumbar disc herniation in the Chinese Han population. Eur. Spine J..

[69] Schistad E.I., Jacobsen L.M., Roe C., Gjerstad J. (2014). The interleukin-1alpha gene C > T polymorphism rs1800587 is associated with increased pain intensity and decreased pressure pain thresholds in patients with lumbar radicular pain. Clin. J. Pain.

[70] Videman T., Saarela J., Kaprio J., Näkki A., Levälahti E., Gill K., Peltonen L., Battié M.C. (2009). Associations of 25 structural, degradative, and inflammatory candidate genes with lumbar disc desiccation, bulging, and height narrowing. Arthritis Rheum..

[71] Puren A.J., Fantuzzi G., Dinarello C.A. (1999). Gene expression, synthesis, and secretion of interleukin 18 and interleukin 1beta are differentially regulated in human blood mononuclear cells and mouse spleen cells. Proc. Natl. Acad. Sci. USA.

[72] Cavanaugh J.M. (1995). Neural mechanisms of lumbar pain. Spine.

[73] Rannou F., Corvol M.T., Hudry C., Anract P., Dumontier M., Tsagris L., Revel M., Poiraudeau S., Serge M.D. (2000). Sensitivity of anulus fibrosus cells to interleukin 1 beta. Comparison with articular chondrocytes. Spine.

[74] Doita M., Kanatani T., Ozaki T., Matsui N., Kurosaka M., Yoshiya S. (2001). Influence of macrophage infiltration of herniated disc tissue on the production of matrix metalloproteinases leading to disc resorption. Spine.

[75] Valdes A.M., Spector T.D., Doherty S., Wheeler M., Hart D.J., Doherty M. (2009). Association of the DVWA and GDF5 polymorphisms with osteoarthritis in UK populations. Ann. Rheum. Dis..

[76] Chapman K., Takahashi A., Meulenbelt I., Watson C., Rodríguez-López J., Egli R., Tsezou A., Malizos K.N., Kloppenburg M., Shi D. (2008). A meta-analysis of European and Asian cohorts reveals a global role of a functional SNP in the 5′ UTR of GDF5 with osteoarthritis susceptibility. Hum. Mol. Genet..

[77] Williams F.M., Popham M., Hart D.J., De Schepper E., Bierma-Zeinstra S., Hofman A., Uitterlinden A.G., Arden N.K., Cooper C., Spector T.D. (2011). GDF5 single-nucleotide polymorphism rs143383 is associated with lumbar disc degeneration in Northern European women. Arthritis Rheum..

[78] Zhang L., Stuber F., Stamer U.M. (2013). Inflammatory mediators influence the expression of nociceptin and its receptor in human whole blood cultures. PLoS ONE.

[79] Mu J., Ge W., Zuo X., Chen Y., Huang C. (2013). Analysis of association between IL-1beta, CASP-9, and GDF5 variants and low-back pain in Chinese male soldier: Clinical article. J. Neurosurg. Spine.

[80] Williams F.M., Bansal A.T., van Meurs J.B., Bell J.T., Meulenbelt I., Suri P., Rivadeneira F., Sambrook P.N., Hofman A., Bierma-Zeinstra S. (2013). Novel genetic variants associated with lumbar disc degeneration in northern Europeans: A meta-analysis of 4600 subjects. Ann. Rheum. Dis..

[81] Liu C.F., Lefebvre V. (2015). The transcription factors SOX9 and SOX5/SOX6 cooperate genome-wide through super-enhancers to drive chondrogenesis. Nucleic Acids Res..

[82] Smits P., Li P., Mandel J., Zhang Z., Deng J.M., Behringer R.R., De Crombrugghe B., Lefebvre V. (2001). The transcription factors L-Sox5 and Sox6 are essential for cartilage formation. Dev. Cell.

[83] Liu C.F., Samsa W.E., Zhou G., Lefebvre V. (2017). Transcriptional control of chondrocyte specification and differentiation. Semin. Cell Dev. Biol..

[84] Smits P., Lefebvre V. (2003). Sox5 and Sox6 are required for notochord extracellular matrix sheath formation, notochord cell survival and development of the nucleus pulposus of intervertebral discs. Development.

[85] Rodriguez-Fontenla C., Calaza M., Evangelou E., Valdes A.M., Arden N., Blanco F.J., Carr A., Chapman K., Deloukas P., Doherty M. (2014). Assessment of osteoarthritis candidate genes in a meta-analysis of nine genome-wide association studies. Arthritis Rheumatol..

[86] Bjornsdottir G., Benonisdottir S., Sveinbjornsson G., Styrkarsdottir U., Thorleifsson G., Walters G.B., Bjornsson A., Olafsson I.H., Ulfarsson E., Vikingsson A. (2017). Sequence variant at 8q24.21 associates with sciatica caused by lumbar disc herniation. Nat. Commun..

[87] Truumees E. (2015). A history of lumbar disc herniation from Hippocrates to the 1990s. Clin. Orthop. Relat. Res..

[88] Chou D., Samartzis D., Bellabarba C., Patel A., Luk K., Kisser J.M.S., Skelly A.C. (2011). Degenerative magnetic resonance imaging changes in patients with chronic low back pain: A systematic review. Spine.

[89] Endean A., Palmer K.T., Coggon D. (2011). Potential of magnetic resonance imaging findings to refine case definition for mechanical low back pain in epidemiological studies: A systematic review. Spine.

[90] Zhang Y., Fukui N., Yahata M., Katsuragawa Y., Tashiro T., Ikegawa S., Lee M.T.M. (2016). Genome-wide DNA methylation profile implicates potential cartilage regeneration at the late stage of knee osteoarthritis. Osteoarthr. Cartil..

[91] Zhang Y., Fukui N., Yahata M., Katsuragawa Y., Tashiro T., Ikegawa S., Lee M.T.M. (2016). Identification of DNA methylation changes associated with disease progression in subchondral bone with site-matched cartilage in knee osteoarthritis. Sci. Rep..

[92] Burston J.J., Sagar D.R., Shao P., Bai M., King E., Brailsford L., Turner J.M., Hathway G., Bennett A.J., Walsh D.A. (2013). Cannabinoid CB2 receptors regulate central sensitization and pain responses associated with osteoarthritis of the knee joint. PLoS ONE.

[93] Rudd R.A., Seth P., David F., Scholl L. (2016). Increases in Drug and Opioid-Involved Overdose Deaths—United States, 2010–2015. Morb. Mortal. Wkly. Rep..

[94] Kolodny A., Courtwright D.T., Hwang C.S., Kreiner P., Eadie J.L., Clark T.W., Alexander G.C. (2015). The prescription opioid and heroin crisis: A public health approach to an epidemic of addiction. Annu. Rev. Public Health.

[95] Knezevic N.N., Tverdohleb T., Knezevic I., Candido K.D. (2018). The Role of Genetic Polymorphisms in Chronic Pain Patients. Int. J. Mol. Sci..

[96] Meloto C.B., Benavides R., Lichtenwalter R.N., Wen X., Tugarinov N., Zorina-Lichtenwalter K., Chabot-Dore A.-J., Piltonen M.H., Cattaneo S., Verma V. (2018). Human pain genetics database: A resource dedicated to human pain genetics research. Pain.

[97] Nelson D.R., Zeldin D.C., Hoffman S.M., Maltais L.J., Wain H.M., Nebert D.W. (2004). Comparison of cytochrome P450 (CYP) genes from the mouse and human genomes, including nomenclature recommendations for genes, pseudogenes and alternative-splice variants. Pharmacogenetics.

[98] Preissner S.C., Hoffmann M.F., Preissner R., Dunkel M., Gewiess A., Preissner S. (2013). Polymorphic cytochrome P450 enzymes (CYPs) and their role in personalized therapy. PLoS ONE.

[99] Yiannakopoulou E. (2013). Pharmacogenomics of acetylsalicylic acid and other nonsteroidal anti-inflammatory agents: Clinical implications. Eur. J. Clin. Pharmacol..

[100] Theken K.N., Lee C.R., Gong L., Caudle K.E., Formea C.M., Gaedigk A., Klein T.E., Agúndez J.A., Grosser T. (2020). Clinical Pharmacogenetics Implementation Consortium Guideline (CPIC) for CYP2C9 and Nonsteroidal Anti-Inflammatory Drugs. Clin. Pharmacol. Ther..

[101] Pilotto A., Seripa D., Franceschi M., Scarcelli C., Colaizzo D., Grandone E., Niro V., Andriulli A., Leandro G., Di Mario F. (2007). Genetic susceptibility to nonsteroidal anti-inflammatory drug-related gastroduodenal bleeding: Role of cytochrome P450 2C9 polymorphisms. Gastroenterology.

[102] Crews K.R., Gaedigk A., Dunnenberger H.M., Klein T.E., Shen D.D., Callaghan J.T., Kharasch E.D., Skaar T.C. (2012). Clinical Pharmacogenetics Implementation Consortium (CPIC) guidelines for codeine therapy in the context of cytochrome P450 2D6 (CYP2D6) genotype. Clin. Pharmacol. Ther..

[103] Dagostino C., Allegri M., Napolioni V., D’Agnelli S., Bignami E., Mutti A., Van Schaik R.H. (2018). CYP2D6 genotype can help to predict effectiveness and safety during opioid treatment for chronic low back pain: Results from a retrospective study in an Italian cohort. Pharmgenomics Pers. Med..

[104] Batistaki C., Chrona E., Kostroglou A., Kostopanagiotou G., Gazouli M. (2020). CYP2D6 Basic Genotyping of Patients with Chronic Pain Receiving Tramadol or Codeine. A Study in a Greek Cohort. Pain Med..

[105] Boswell M.V., Stauble M.E., Loyd G.E., Langman L., Ramey-Hartung B., Baumgartner R.N., Tucker W.W., Jortani S.A. (2013). The role of hydromorphone and OPRM1 in postoperative pain relief with hydrocodone. Pain Physician.

[106] Margarit C., Roca R., Inda M.D., Muriel J., Ballester P., Moreu R., Conte A.L., Nuñez A., Morales M., Peiró A.M. (2019). Genetic Contribution in Low Back Pain: A Prospective Genetic Association Study. Pain Pract..

[107] Rodieux F., Piguet V., Berney P., Desmeules J., Besson M. (2015). Pharmacogenetics and analgesic effects of antidepressants in chronic pain management. Pers. Med..

[108] Samer C.F., Lorenzini K.I., Rollason V., Daali Y., Desmeules J.A. (2013). Applications of CYP450 testing in the clinical setting. Mol. Diagn. Ther..

[109] Staddon S., Arranz M.J., Mancama D., Mata I., Kerwin R.W. (2002). Clinical applications of pharmacogenetics in psychiatry. Psychopharmacology.

[110] Bertilsson L., Dahl M.L., Dalen P., Al-Shurbaji A. (2002). Molecular genetics of CYP2D6: Clinical relevance with focus on psychotropic drugs. Br. J. Clin. Pharmacol..

[111] Swen J.J., Nijenhuis M., de Boer A., Grandia L., Maitland-van der Zee A.H., Mulder H., Rongen G.A.P.J.M., Van Schaik R.H.N., Schalekamp T., Touw D.J. (2011). Pharmacogenetics: From bench to byte—An update of guidelines. Clin. Pharmacol. Ther..

[112] Chang H.H., Chou C.H., Yang Y.K., Lee I.H., Chen P.S. (2015). Association between ABCB1 Polymorphisms and Antidepressant Treatment Response in Taiwanese Major Depressive Patients. Clin. Psychopharmacol. Neurosci..

[113] Singh A.B., Bousman C.A., Ng C.H., Byron K., Berk M. (2012). ABCB1 polymorphism predicts escitalopram dose needed for remission in major depression. Transl. Psychiatry.

[114] Suwala J., Machowska M., Wiela-Hojenska A. (2019). Venlafaxine pharmacogenetics: A comprehensive review. Pharmacogenomics.

[115] Ma Y., Wang C., Luo S., Li B., Wager T.D., Zhang W., Rao Y., Han S. (2016). Serotonin transporter polymorphism alters citalopram effects on human pain responses to physical pain. Neuroimage.

[116] Winchell G.A., King J.D., Chavez-Eng C.M., Constanzer M.L., Korn S.H. (2002). Cyclobenzaprine pharmacokinetics, including the effects of age, gender, and hepatic insufficiency. J. Clin. Pharmacol..

[117] Mestres J., Seifert S.A., Oprea T.I. (2011). Linking pharmacology to clinical reports: Cyclobenzaprine and its possible association with serotonin syndrome. Clin. Pharmacol. Ther..

[118] Perucca E. (2006). Clinically relevant drug interactions with antiepileptic drugs. Br. J. Clin. Pharmacol..

[119] Ben-Menachem E. (2004). Pregabalin pharmacology and its relevance to clinical practice. Epilepsia.

[120] Honarmand A., Safavi M., Zare M. (2011). Gabapentin: An update of its pharmacological properties and therapeutic use in epilepsy. J. Res. Med. Sci..

[121] Koepsell H. (2013). The SLC22 family with transporters of organic cations, anions and zwitterions. Mol. Asp. Med..

[122] Koepsell H., Lips K., Volk C. (2007). Polyspecific organic cation transporters: Structure, function, physiological roles, and biopharmaceutical implications. Pharm. Res..

[123] Yamamoto P.A., Benzi J.R.L., Azeredo F.J., Dach F., Ianhez Junior E., Zanelli C.F., De Moraes N.V. (2019). Pharmacogenetics-based population pharmacokinetic analysis of gabapentin in patients with chronic pain: Effect of OCT2 and OCTN1 gene polymorphisms. Basic Clin. Pharmacol. Toxicol..

[124] Pergolizzi J.V., Labhsetwar S.A., Puenpatom R.A., Joo S., Ben-Joseph R.H., Summers K.H. (2011). Prevalence of exposure to potential CYP450 pharmacokinetic drug-drug interactions among patients with chronic low back pain taking opioids. Pain Pract..

[125] Pergolizzi J.V., Ma L., Foster D.R., Overholser B.R., Sowinski K.M., Taylor R., Summers K.H. (2014). The prevalence of opioid-related major potential drug-drug interactions and their impact on health care costs in chronic pain patients. J. Manag. Care Spec. Pharm..

[126] Verbeurgt P., Mamiya T., Oesterheld J. (2014). How common are drug and gene interactions? Prevalence in a sample of 1143 patients with CYP2C9, CYP2C19 and CYP2D6 genotyping. Pharmacogenomics.

[127] Malki M.A., Pearson E.R. (2020). Drug-drug-gene interactions and adverse drug reactions. Pharm. J..

[128] Storelli F., Desmeules J., Daali Y. (2019). Physiologically-Based Pharmacokinetic Modeling for the Prediction of CYP2D6-Mediated Gene-Drug-Drug Interactions. CPT Pharmacomet. Syst. Pharmacol..

[129] Trescot A.M., Faynboym S. (2014). A review of the role of genetic testing in pain medicine. Pain Physician.

